# Can Stem Cells Beat COVID-19: Advancing Stem Cells and Extracellular Vesicles Toward Mainstream Medicine for Lung Injuries Associated With SARS-CoV-2 Infections

**DOI:** 10.3389/fbioe.2020.00554

**Published:** 2020-05-26

**Authors:** Wojciech Chrzanowski, Sally Yunsun Kim, Lana McClements

**Affiliations:** ^1^Faculty of Medicine and Health, Sydney School of Pharmacy, Sydney Nano Institute, The University of Sydney, Camperdown, NSW, Australia; ^2^National Heart and Lung Institute, Imperial College London, London, United Kingdom; ^3^Faculty of Science, School of Life Sciences, University of Technology Sydney, Sydney, NSW, Australia

**Keywords:** stem cell, lung injuries, Coronavirus (2019-nCoV), SARS-CoV-2, stem cell therapy, extracellular vesicles

## Abstract

A number of medicines are currently under investigation for the treatment of COVID-19 disease including anti-viral, anti-malarial, and anti-inflammatory agents. While these treatments can improve patient's recovery and survival, these therapeutic strategies do not lead to unequivocal restoration of the lung damage inflicted by this disease. Stem cell therapies and, more recently, their secreted extracellular vesicles (EVs), are emerging as new promising treatments, which could attenuate inflammation but also regenerate the lung damage caused by COVID-19. Stem cells exert their immunomodulatory, anti-oxidant, and reparative therapeutic effects likely through their EVs, and therefore, could be beneficial, alone or in combination with other therapeutic agents, in people with COVID-19. In this review article, we outline the mechanisms of cytokine storm and lung damage caused by SARS-CoV-2 virus leading to COVID-19 disease and how mesenchymal stem cells (MSCs) and their secreted EVs can be utilized to tackle this damage by harnessing their regenerative properties, which gives them potential enhanced clinical utility compared to other investigated pharmacological treatments. There are currently 17 clinical trials evaluating the therapeutic potential of MSCs for the treatment of COVID-19, the majority of which are administered intravenously with only one clinical trial testing MSC-derived exosomes via inhalation route. While we wait for the outcomes from these trials to be reported, here we emphasize opportunities and risks associated with these therapies, as well as delineate the major roadblocks to progressing these promising curative therapies toward mainstream treatment for COVID-19.

## Understanding Coronaviruses and Their Impact on Health

A new strain of coronavirus, SARS-CoV-2 (severe acute respiratory syndrome coronavirus 2, as named by the International Committee on Taxonomy of Viruses) discovered in Wuhan, China, in December 2019, has caused a global disruption and COVID-19 pandemic. SARS-CoV-2 is classified as *Betacoronavirus*. In general, coronavirus is the name of viruses that belong to the family *Coronaviridae*. These are classified into four categories: *Alphacoronavirus, Betacoronavirus, Gammacoronavirus*, and *Deltacoronavirus*. Alpha- and betacoronaviruses infect mammals, gammacoronaviruses infect avian species, and deltacoronaviruses infect both mammalian and avian species (Li, 2016). Coronaviruses are typically spherical, fatty enveloped viruses, which encapsulate large single-stranded RNA genomes, notably, the largest genome among all RNA viruses, typically ranging from 27 to 32 kb. The genome is packed inside a helical capsid formed by the nucleocapsid protein (N) and further surrounded by the envelope.

**Graphical Abstract:**
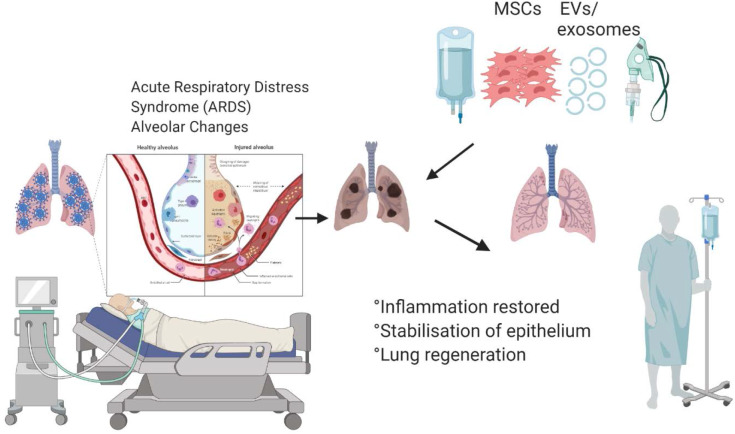
Mesenchymal stem cells and their secreted extracellular vesicles are capable of abrogating inflammation and inducing lung regeneration, which can improve recovery of the patients hospitalized with COVID-19.

Coronaviruses have characteristic clove-shape spikes (“corona”) protruding from the surface. The spikes are protein complexes that virus uses to bind to a receptor (receptor-binding subunit S1) and mediate entry into host cells (a membrane-fusion subunit S2). Upon binding virus fuses with the human cell membrane, allowing the genome of the virus to enter the cell and begin replication. The receptor-binding domain of SARS-CoV-2 spikes are closely related to those of SARS-CoVs (73.8–74.9% amino acid identity) and SARS-like CoVs (75.9–76.9% amino acid identity) (Wu et al., 2020). SARS-CoV-2 uses the human angiotensin I converting enzyme 2 (ACE2) receptor for cell entry (Lu et al., 2020), which could potentially facilitate human-to-human transmission. In addition to mediating virus entry, the spike feature is a critical determinant of viral host range and tissue tropism and a cause of host immune responses.

**Figure 1:**
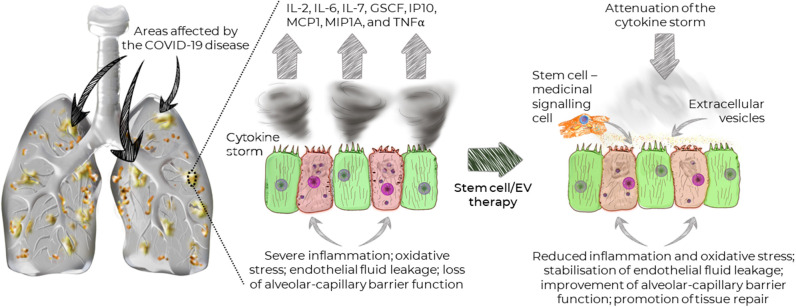
Widespread distribution of the areas affected by COVID-19 and the effects of stem cells and extracellular vesicles on the recovery of lung function.

Since the ACE2 receptor is widely distributed on the human alveolar type II cells and capillary endothelium (Hamming et al., 2004), lungs are particularly susceptible to the SARV-CoV-2 infections. Indeed, recent studies demonstrated that the disease could affect a large portion of the lungs causing widespread damage (Figure 1), which subsequently may lead to permanent changes to the lung function. Recent MRI study of a 59-year old male patient diagnosed with COVID-19 showed that the disease is not confined to any particular area, but that it can spread across the entire lungs (Figure 1; Mortman, 2020). However, this is not caused directly by the virus, but rather the host immune response leading to overwhelming cytokine storm in the lungs. The overexpression of cytokines such as IL-2, IL-6, IL-7, GSCF, IP10, MCP1, MIP1A, and TNFα is followed by edema, which impairs oxygen exchange, and may lead to acute respiratory distress syndrome (ARDS) causing potential acute cardiac injury, secondary infection (Huang et al., 2020), and death.

Most recent observations suggest that the devastating complication of COVID-19 may not be a typical pneumonia or ARDS, but rather a dysfunction in blood oxygenation. This statement is supported by the fact that over 80% of COVID-19 patients placed on ventilators in New York City have died (Baker, 2020). According to Dr. Cameron Kyle-Sidell (Maimonides Medical Center, New York, March 31, 2020), COVID-19 patients show symptoms associated with high altitude without adaptation response (Gattinoni et al., 2020a,b). Clinically these cases resemble more closely high-altitude sickness characterized by decompression pulmonary sickness or high-altitude pulmonary edema (HAPE) with distinctive features of severe hypoxemia often associated with near normal respiratory system compliance.

Since cytokines and inflammation play dominant role in the development of COVID-19-induced lung damage, immunological therapies capable of attenuating cytokine storm may be the key treatment options. However, traditional immunomodulatory capacity of common immunotherapeutics are targeted at one or two factors, which may not induce a strong enough response.

Mesenchymal stem/stromal cells (MSCs) have demonstrated potent and broad immunomodulatory and anti-inflammatory capacity (Abdi et al., 2008; Wada et al., 2013). Therefore, MSC-based therapy could potentially be an effective treatment for COVID-19. Notably, due to their regenerative properties (Mahla, 2016), MSCs can promote the repair of damaged tissue, in order to prevent long-term lung damage inflicted by COVID-19. Importantly, MSCs have been shown to stabilize endothelial fluid leakage and maintain alveolar-capillary barrier function, which is vital to sustain or decrease lung permeability due to inflammation, thus attenuating the development of interstitial lung edema (Bhattacharya and Matthay, 2013).

## Are We All Affected the Same Way by Coronavirus?

Coronavirus-related infections can cause a variety of illnesses, which range from asymptomatic or mild such as a common cold, to severe including Severe Acute Respiratory Syndrome (SARS), Middle East Respiratory Syndrome (MERS), and now, COVID-19. The severity of these infections also varies widely between age-groups and different ethnic populations. Older people are at increased risk of acquiring the infection and are likely to develop severe symptoms culminating in death.

Interestingly, there appears to also be gender disparity in the number of acquired cases of COVID-19 with higher percentage of men (~60%) than women being infected, as was first reported in China (Li et al., 2020); other countries have also reported higher case fatality rate (CFR) in men^1^. Similar observations were reported in a meta-analysis in relation to the MERS-CoV, where the number of infected men was double that of females (Badawi and Ryoo, 2016). Epidemiological studies tracing SARS-CoV showed that SARS-CoV was also more prevalent in men and associated with increased CFR (Karlberg, 2004). A number of proposed explanations for these gender differences in terms of both the incidence and CFR of MERS-CoV, SARS-CoV, and now COVID-19 infections, include past smoking history, work-environmental factors, different treatment regimens, and underlying biological differences such as gender-specific innate and adaptive immune responses (Karlberg, 2004; Klein and Flanagan, 2016). Clear biological differences between male and female gender include the presence of different steroid hormones and two instead of one X-chromosomes in females, which influences the number of immune response X-linked genes and genetic susceptibility to viral infections (Klein and Flanagan, 2016). Notably, experiments in mice, which seem to represent responses in humans well in this particular study, demonstrated greater susceptibility of male mice to enhanced viral titres, vascular leakage, and alveolar edema, compared to age-matched female mice infected with SARS-CoV (Channappanavar et al., 2017). This was attributed to differential immune cells and inflammatory responses including inflammatory monocyte macrophages, neutrophils, T- and B-cells. Notably, in this pre-clinical study, sex-dependent differences in susceptibility to SARS-CoV infection became more prominent with advancing age. In fact, once female mice underwent ovariectomy or were administered estrogen receptor antagonist treatment, the mortality associated with SARS-CoV infection increased, suggesting a protective effect of estrogen receptor signaling. Although experimental and mechanistic data is lacking in relation to the novel COVID-19 sex-specific differences, it is likely that similar factors and biological differences will be applicable as with MERS-CoV and SARS-CoV. It is important for these differences to be clearly elucidated particularly in the context of the treatment response, with certain treatments potentially being more effective in a specific gender group. Nevertheless, other confounding factors need to be taken into the account such as age, smoking, comorbidities and work-environmental circumstances.

## Current Treatments Are Palliative Only

Despite over 300 active and recruiting clinical trials and a number of trials already completed, there is still no robust evidence that any of the investigated therapeutics are effective as treatments for COVID-19 disease (Channappanavar et al., 2017). Equally, there is no evidence to support prophylactic treatment either (Sanders et al., 2020). However, there are only 29 trials in adult patients with placebo-controlled arm (Channappanavar et al., 2017). Various types of pharmacological treatments are currently under investigation including anti-viral, anti-malarial and anti-inflammatory agents. These therapies generally target the following processes: (1) the entry of the virus into host cells, (2) multiplication of the viral genetic material, and (3) immune response/inflammation. Most of these agents have been previously used as treatments for SARS-CoV and MERS-CoV, however the overall conclusions of the meta-analyses published in 2006 and 2018, respectively, did not support the use of any particular regimen. In relation to the meta-analysis of SARS-CoV treatments, the authors systematically reviewed 54 treatment studies, 15 *in vitro* studies and three ARDS studies (Stockman et al., 2006). Although the combination of ribavirin and interferon-based (IFN) treatments appears the most effective for MERS (Morra et al., 2018), this needs to be confirmed in randomized placebo-controlled trial settings. In terms of vaccines, there are at least 115 vaccine candidates in development with a number of these already initiated in human trials, however we expect vaccines to be available to people under emergency use only in early 2021 (Callaway, 2020; Thanh Le et al., 2020).

Overall, there are a number of concerns in relation to the design of various trials and interpretation of the data investigating different pharmacological agents for the treatment of COVID-19. Some of these limitations include small cohort sizes, no placebo control arm, lack of considerations for gender, comorbidities, concurrent treatments, route of drug delivery, primary outcomes lacking effects on the viral load or suppression, and adverse drug effects. Whilst most of these therapies represent supportive and symptomatic care, there are a number of adjunctive therapies such as corticosteroids, immunomodulatory, and immunoglobulin agents that have been investigated with limited results. In particular, corticosteroids are not recommended for the management of COVID-19 because of the associated adverse effects, which potentially include increased viral load, secondary infections and complications, similarly to what was observed previously in influenza, SARS-CoV and MERS-CoV infections (Russell et al., 2020). Potential benefits in severe COVID-19 cases are emerging with IL-6 monoclonal antibody, Tocilizumab, and the use of convalescent plasma or hyperimmune immunoglobulins, however better designs and further trials are needed for this to be established (Chen L. et al., 2020; Fu et al., 2020). Nevertheless, none of these therapies are capable of lung tissue repair and regeneration, particularly in those patients with complications such as ARDS, which is why the use of stem cell-based therapies could be beneficial in COVID-19 patients with respiratory complications.

## Are Stem Cells a Solution to COVID-19 Crisis?

MSCs could be the most promising candidate for the treatment of SARS-CoV-2 infections (Table 1). Since the key for the treatment of SARS-CoV-2 infection lies in the management of the cytokine storm in the lungs, MSCs are well-suited considering their main mechanism of action is through their immunomodulatory and anti-inflammatory properties (Fatima et al., 2017). The safety profile and efficacy of MSCs are well-established based on the results from a number of completed clinical studies investigating the therapeutic potential of these therapies in lung diseases such as ARDS (Matthay et al., 2019; Chen J. et al., 2020) and bronchopulmonary dysplasia (Namba, 2019), cardiovascular diseases (Kim et al., 2015; Suvakov et al., 2020), diabetes (Thakkar et al., 2015; Cho et al., 2018), and spinal cord injury (Xu and Yang, 2019).

**Table 1:** Selected clinical studies using stem cells for the treatment of SARS-CoV-2 infection.

**Cell type**	**Dosage**	**Number of patients**	**Outcome**	**Stage of study**	**Reference or NCT numbers^#^**
MSCs (tissue source unspecified)	Single dose of 1 × 10^6^ cells per kg, IV	7 patients with severe COVID-19 pneumonia	Regulation of inflammatory response (Decreased plasma C-reaction protein, reduced cytokine-secreting immune cells, reduced TNF-α, increased IL-10 and VEGF)	Complete	Leng et al., 2020
Adipose tissue-derived MSCs	Two serial doses of 1.5 × 10^6^ cells per kg, IV	Estimated: 100 patients	N/A	Phase 2; Not yet recruiting	NCT04348461
Dental pulp stem cells	3 × 10^7^ cells IV on day 1, 4, and 7	Estimated: 20 patients	N/A	Phase 1 clinical trial; recruiting	NCT04336254
Dental pulp stem cells	1 × 10^6^ cells per kg IV on day 1, 3, and 7	Estimated: 24 patients	N/A	Early Phase 1; Not yet recruiting	NCT04302519
Wharton's Jelly MSCs	3 doses of 1 × 10^6^ cells per kg IV, 3 days apart from each other	Estimated: 5 patients	N/A	Phase 1 clinical trial; recruiting	NCT04313322
MSCs (tissue source unspecified)	3 × 10^7^ cells IV on day 0, 3, and 6	Estimated: 20 patients	N/A	Phase 1 clinical trial; recruiting	NCT04252118
Umbilical cord MSCs	9.9 × 10^7^ cells IV on day 1, 3, 5, and 7	Estimated: 10 patients	N/A	Phase 2 clinical trial; recruiting	NCT04269525
Umbilical cord MSCs	0.5 × 10^6^ cells per kg body weight IV on day 1, 3, 5, and 7	Estimated: 48 patients	N/A	Not yet recruiting	NCT04273646
Bone Marrow MSCs	Single dose of 1 × 10^6^ cells per kg, IV	Estimated: 20 patients	N/A	Phase 1 clinical trial; Not yet recruiting	NCT04346368
Human embryonic stem cells derived matrix-regulatory cells	3 cohorts who receive doses of 3, 5, or 10 million cells per kg body weight IV	Estimated: 9 patients	N/A	Phase 1 clinical trial; recruiting	NCT04331613

# *NCT numbers refer to ClinicalTrials.gov Identifier numbers*.

Other types of stem cells investigated for potential treatment of SARS-CoV-2 infections include genetically engineered human induced pluripotent stem cells (iPSC). A recent study presented a deleterious effect on the cells *in vitro* when iPSCs were exposed to SARS-CoV-2, where the pluripotency of iPSCs was lost leading to fibroblast-like phenotype (Zebin et al., 2020). Therefore, evidence-based selection of stem cell type for the treatment of COVID-19 is critical for safety and efficacy.

## Advancing Stem Cells to Mainstream Medicines for COVID-19

Currently, there are 17 clinical trials investigating the therapeutic potential of MSCs in COVID-19 patients that are registered on clinicaltrials.gov website; most of these trials are either recruiting patients or have not yet started the recruitment. The vast majority of the trials are selecting patients with COVID-19 and pneumonia, and utilizing allogeneic bone-marrow or umbilical cord-derived MSCs transplanted intravenously on three different occasions (Table 1). Approximately 50% of the trials are being carried out in China. Three trials (NCT03042143 already recruiting in Northern Ireland, United Kingdom; NCT04333368 planned in France and NCT04345601 in the United States of America, not yet recruiting) aim to investigate the therapeutic potential of MSCs in SARS-CoV-2-induced ARDS. MSCs have already been investigated in ARDS both in the pre-clinical (Curley et al., 2013) and clinical settings (Wilson et al., 2015), which demonstrated the ability of MSCs to promote distal lung epithelial repair, albeit potentially limited in the presence of hypercapnic acidosis (Fergie et al., 2019).

**Figure 2:**
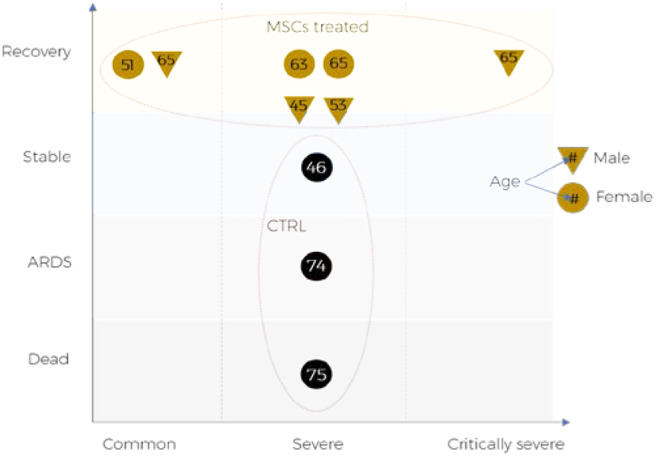
Outcomes of MSCs treatment for COVID-19 patients reported in pilot clinical trial: ChiCTR2000029990.

Whilst a number of MSC-related trials in COVID-19 patients with respiratory complications are underway, limited reports are available that these treatments have the potential to improve recovery and survival of these patients. Some encouraging data is emerging, for example in a recent study, which assessed 7 patients transplanted with intravenous MSCs (1 × 10^6^ per kg) and 3 patients in the placebo controlled group albeit consisting of all female patients, showed significant improvement in pulmonary function and symptoms as well as substantial reduction in inflammation compared to the placebo controlled group (Figure 2).

Despite the lack of convincing evidence in support of MSC-based therapies for respiratory complications of COVID-19, a number of promising trials are currently underway, which could revolutionize the treatment prospects for severe COVID-19 patients. Regardless of the urgency of the need for MSC-based therapies for COVID-19, it is critical that the production of MSCs is in compliance with good manufacturing practices (GMP) and follows strict regulations prior to being approved for the use in humans. The use of unauthorized and unapproved stem cell therapies not validated through stringent multicenter clinical trials should be strongly discouraged. At the same time current findings imply that there is a need for globally coordinated approach and support to conduct multicenter clinical trials to demonstrate safety and effectiveness of various types of stem cells to treat COVID-19-induced health complications. It also suggests that there is a need in biomedical research and development to establish the most effective stem cell types that are ideally suited for the treatment of aforementioned complications. These developments will also require (a) GMP compliant technologies to mass produce stem cells, and (b) testing platforms that mimic human pathophysiology (e.g., 3D bioprinted organoids, organ-on-chip) to allow high-throughput screening and rapid testing of stem cells safety and efficacy.

Extracellular vesicles (EVs) are emerging as an attractive alternative to the whole cell–based therapy. EVs have several advantages compared to the whole cell therapy including lower risk of tumorigenic effects, lower susceptibility to damage by hostile disease microenvironment and possibility for long-term storage. The long-term storage is fundamental to make the treatment accessible in developing countries and it circumvents the need to have expensive GMP cell manufacturing facilities on-site. Nevertheless, production of EVs must follow the same strict guidelines that apply to stem cells and any EV-based therapy needs to be approved by the governing bodies after being tested in clinical trials to demonstrate the safety and efficacy.

## Factors Affecting the Quality of MSC

The successful translation of MSC-based therapies into the clinic for treatment of COVID-19-induced lung injuries is dependent on a number of factors. In addition to desirable therapeutic effects, it is also important to understand the mechanism of action and the influence of various *in vivo* environments on the integrity of these cells. Stem cells can be environmentally preconditioned by certain stimuli such as hypoxia or ischemia, which can induce certain signaling pathways to improve engraftment, survival, and function of transplanted cells in harsh environments within the injured lung (Sart et al., 2014; Kim et al., 2015; Saparov et al., 2016). Other methods of preconditioning stem cells include the formation of three-dimensional stem cell aggregates that lead to enhanced extracellular matrix secretion, anti-inflammatory properties and cell survival (Bartosh et al., 2010; Sart et al., 2014). Pre-treatment of MSCs with various pharmacological factors can, in fact, improve therapeutic effects and tissue repair capabilities (Hu and Li, 2018).

Furthermore, bioengineering approaches such as the use of bioreactors can overcome the limitations of a large scale production of stem cells necessary for transplantation and maintenance of the stemness during and after delivery to patients (Madl et al., 2018). Therefore, these technologies can accelerate the transition from bench to bedside without compromising the quality of the stem cells reaching patients. For example, tuneable materials can be used as an artificial niche to expand or differentiate stem cells to mature cell types (Yang et al., 2014; Madl et al., 2017). Materials such as hydrogels used as carriers for stem cells can also lead to enhanced regenerative response by regulating fate and activity of transplanted stem cells (Engler et al., 2006; Chaudhuri et al., 2016). These approaches are currently less widely used for pulmonary applications compared to other target organs and thus future investigations will lead to innovative solutions.

## Is the Treatment Efficacy Dependent on the Delivery Route?

In the majority of clinical trials investigating the treatment of SARS-CoV-2 infections so far, MSCs are delivered via the intravenous route. Since intravenous route does not specifically target lungs, the inhalation route that delivers cells directly to lungs could be more effective. However, uniform delivery of cells to lungs through inhalation is technically challenging (Kim et al., 2016; Kim and Chrzanowski, 2019). Strong evidence is emerging to suggest that the therapeutic potential of MSCs is attributed mostly to their secreted EVs via paracrine effects, and MSCs could also be referred to as medicinal signaling cells (Bjørge et al., 2018; Kim et al., 2019). Delivery of EVs to lungs is feasible and exogenous EVs can facilitate prompt activity directly at the injured site. There is a pilot clinical study that intends to deliver MSC-derived exosomes via the inhalation route to patients with severe pneumonia arising from SARS-CoV-2 infection (NCT04276987). In this clinical trial, the main advantage of administering MSC-derived exosomes via inhalation route compared to the intravenous administration is the prevention of their aggregation within the injured microcirculation. In addition, MSC-derived exosomes have other advantages over MSCs including lower or no risk of mutagenicity and oncogenicity as well as the storage stability of several weeks or months facilitating less urgent transportation and administration. A recent study using animal models of pulmonary fibrosis also demonstrated therapeutic potential of inhaled lung spheroid cell-secretome and exosomes for lung regeneration (Dinh et al., 2020). While the intravenous route is the most widely used often for the ease of administration albeit invasive, the route of cell delivery should be tailored according to the disease in question and patient's circumstances (Kim and Chrzanowski, 2019). As evident from a number of clinically available inhaled medications for chronic lung disease, the inhalation route of delivering therapeutics to the lungs is a more direct route with lower incidence of adverse effects, compared to the intravenous route. Nevertheless, the environment in the hospital has to be appropriately managed for inhaled administration of a treatment in COVID-19 patients. A number of studies have demonstrated the feasibility of delivering stem cells via spray for direct pulmonary delivery with high viability (Kardia et al., 2016; Kim et al., 2016; Skolasinski et al., 2020). Inhalation route of stem cell administration is an opportunity for efficient delivery of stem cells directly to the lungs, yet it remains an under-explored research area in the field.

## The Future of Stem Cells for the Treatment of SARS-CoV-2 and Other Coronaviruses Infections

Although only a limited number of MSC-based clinical trials have been completed to date, in a small number of COVID-19 patients, the prospects of using these cells in the clinical setting to treat and prevent COVID-19-induced lung damage appear promising. Successfully completed Phase I trials for the treatment of ARDS and emerging promising preliminary results from current trials will soon shed light on the effectiveness of MSCs for the treatment of SARS-CoV-2. The proposed clinical study that aims to deliver MSC-derived exosomes via the inhalation route to COVID-19 patients (NCT04276987) will potentially be a paradigm shift. In the light of a recent study that demonstrated efficacy of inhaled stem cell-derived therapy in both *ex vivo* and animal models of pulmonary fibrosis (Dinh et al., 2020), the inhalation route due to advantages outlined above, may become more routinely investigated along with the conventional intravenous route. Importantly, regardless of the route of delivery and type of stem cells, larger-scale clinical trials strictly designed with the placebo arm and patient randomization are necessary to achieve successful stem cell-based therapies for the treatment of COVID-19.

## Data Availability Statement

The original contributions presented in the study are included in the article/supplementary materials, further inquiries can be directed to the corresponding author/s.

## Author Contributions

All authors listed have made a substantial, direct and intellectual contribution to the work, and approved it for publication.

## Conflict of Interest

The authors declare that the research was conducted in the absence of any commercial or financial relationships that could be construed as a potential conflict of interest.

## Footnotes

^1^*COVID-19 Sex-Disaggregated Data Tracker*. Global Health 5050.
